# Oxytetracycline-induced inflammatory process without oxidative stress in blue mussels *Mytilus trossulus*

**DOI:** 10.1007/s11356-023-28057-z

**Published:** 2023-06-10

**Authors:** Anna Hallmann, Dagmara Leszczyńska, Aleksandra Czumaj, Justyna Świeżak, Magda Caban, Alicja Michnowska, Katarzyna Smolarz

**Affiliations:** 1 Department of Pharmaceutical Biochemistry, Medical University of Gdańsk, Gdańsk, Poland; 2 Department of Marine Ecosystem Functioning, University of Gdańsk, Gdynia, Poland; 3 Department of Environmental Analysis, Faculty of Chemistry, University of Gdańsk, Gdańsk, Poland

**Keywords:** Antibiotics, Bivalves, Health, Oxidative stress, Phenoloxidase, Immunocompetence

## Abstract

Potentially harmful compounds including pharmaceuticals are commonly found in marine waters and sediments. Amongst those, antibiotics and their metabolites are detected worldwide in various abiotic (at concentrations as high as µg/L) and biotic matrices at ng/gram of tissue, posing a risk to non-target species exposed to them such as blue mussels. Amongst those, oxytetracycline (OTC) belongs to the most detected antibiotics in the marine environment. In this work, we concentrated on studying the potential induction of oxidative stress, activation of cellular detoxification processes (including Phase I and Phase II xenobiotic biotransformation enzymes) and multixenobiotic resistance pumps (Phase III) as well as changes in the aromatisation efficiency in *Mytilus trossulus* exposed to 100 μg/L OTC. Our results show that 100 µg/L OTC concentration did not provoke cellular oxidative stress and did not affect the expression of genes involved in detoxification processes in our model. Moreover, no effect of OTC on aromatisation efficiency was found. Instead, phenoloxidase activity measured in haemolymph was significantly higher in OTC exposed mussels than in those from the control (30.95 ± 3.33 U/L and 17.95 ± 2.75 U/L, respectively). OTC exposed mussels were also characterised by a tissue-dependant activation of major vault protein (MVP) gene expression (1.5 times higher in gills and 2.4 times higher in the digestive system) and a decreased expression of the nuclear factor kappa B-a (NF-κB) gene (3.4 times lower in the digestive system) when compared to those from the control. Additionally, an elevated number of regressive changes and inflammatory responses in tissues such as gills, digestive system and mantle (gonads) was observed underlining the worsening of bivalves’ general health. Therefore, instead of a free-radical effect of OTC, we for the first time describe the occurrence of typical changes resulting from antibiotic therapy in non-target organisms like *M. trossulus* exposed to antibiotics such as OTC.

**Supplementary Information:**

The online version contains supplementary material available at 10.1007/s11356-023-28057-z.

## Introduction

As a result of increasing anthropogenic pressure, a large diversity of potentially harmful compounds is found in marine waters and sediments. These compounds include contaminants of emerging concern comprising various pharmaceutical residues detected at concentration ranges between ng/L and µg/L (Arpin-Pont et al. 2016) in various matrices including biota, water (wastewater, surface water, drinking water, groundwater), sewage sludge and sediment (Vieno et al. 2017). Amongst residues of pharmaceutical compounds present in the marine environment, antibiotics are of particular importance (Kümmerer 2009a, b), as they are widely used to treat and prevent diseases in humans and animals, as well as to increase the growth rate of farmed animals and poultry (Sarmah et al. 2006; Gao et al. 2012). Although the use of antibiotics in feeding for growth promotion has been banned in the European Union since 2006 (Regulation No 1831/2003), in some countries such as Canada, the USA and Korea, their use is still widespread (Sarmah et al. 2006). According to available data from 71 highly populated countries, between 2000 and 2010, the total global antibiotic consumption grew by more than 30%, from approximately 50 billion to 70 billion standard units (Van Boeckel et al. 2015). It has also been estimated that antimicrobial drug consumption will rise by 67% by 2030, and nearly double in Brazil, Russia, India, China and South Africa (Van Boeckel et al. 2015). Additionally, bioaccumulation and biomagnification of these compounds may stimulate the occurrence of one of the biggest public health challenges of our time–antibiotic resistance, directly in the aquatic environment, which may pose danger for higher trophic levels including the human population (Suzuki et al. 2017).

Pharmaceuticals, including antibiotics, enter the environment through the release of raw and treated urban, industrial and agricultural wastewater (Kim and Tanaka 2009; Shelver et al. 2010). This class of drugs and their metabolites are detected in surface waters, groundwater, wastewater and drinking water at concentrations ranging from ng/L to µg/L (Christian et al. 2003; Kim and Tanaka 2009; López-Peñalver et al. 2010) whilst in seawater at concentrations up to µg/L (Arpin-Pont et al. 2016). In general, their highest concentrations occur in marine coastal areas that are under immense pressure due to human activities (industry, urbanisation, maritime transportation). As pharmaceuticals are biologically active compounds, they also exert pressure over species accidentally exposed to them directly in the ambient environment, the so-called non-target species. Due to easy adsorption and high accumulation potential of antibiotics, many non-target marine organisms are highly exposed to this group of drugs (Liu et al. 2017).

Tetracycline antibiotics are a group of broad-spectrum compounds that exhibit antibiotic activity against infections caused by both Gram ( +) and Gram (-) bacteria, as well as mycoplasma, chlamydia and rickettsia (Chopra and Roberts 2001). Due to their low cost of production and high antimicrobial activity, tetracycline antibiotics are one of the main groups of antibiotics used in veterinary medicine, human treatment and agriculture as food additives (Granados-Chinchilla and Rodríguez 2017). Chlortetracycline (CTC), oxytetracycline (OTC) and tetracycline (TC) are the most used worldwide types of tetracycline antibiotics (Halling-Sørensen et al. 1998; Kümmerer 2003). TC is used in aquaculture and veterinary medicine (López-Peñalver et al. 2010), whilst CTC and OTC are food additives used as growth stimulators in, for example, the United States (Yang and Carlson 2003; Jeong et al. 2010). Due to the widespread usage of tetracycline antibiotics, they have been detected in aquatic environments: in surface water at concentrations of up to 44 µg/L (Boxall et al. 2006), in groundwater at concentrations of up to 50 ng/L (Díaz-Cruz and Barceló 2005), and in municipal sewages at concentrations of up to 65 µg/L (Liu et al. 2009b). In seawater, concentration of tetracycline derivatives ranged from 13 to 59 ng/L, whilst the concentrations found in sediment ranged from 6 to 300 ng/kg for samples taken in Laizhou Bay (Weifang, China) (Gao et al. 2018). The generally higher levels of tetracycline antibiotics in surface water (Li et al. 2011), groundwater, wastewater (Jeong et al. 2010; López-Peñalver et al. 2010) domestic sewage, soil and sediments (Liu et al. 2009a) imply that conventional wastewater treatment plants (WWTPs) cannot eliminate them. Yet, WWTPs are characterised by the removal efficiency of tetracycline from 25 to 80% (Daghrir and Drogui 2013; Zhang et al. 2015). Thus, tetracycline concentrations ranging from 0.15 to 0.97 µg/L have been reported in WWTPs waste waters in Canada (Miao et al. 2004) whilst those detected in the USA surface waters were 0.11 (TC), 1.34 (OTC) and 0.15 (CTC) µg/L (Lindsay et al. 2001). In fish farms using OTC, its levels in the sediments were higher whilst the compound persisted for several months (Samuelsen et al. 1992). OTC has also been reported at a concentration of 10.2 μg/g of soft tissue in the blue mussels *Mytilus edulis* collected in the vicinity of an Atlantic salmon farm, where the antibiotic was used as a therapeutic treatment in concentration of 125 mg/kg body weight (Coyne et al. 1997). The presence of tetracycline antibiotics was also recorded in the Baltic Sea region, and specifically in the sediments of the Gulf of Gdańsk near the WWTP ‘Gdańsk Wschód’. In this area, OTC levels ranged from 21 to 625 ng/g dry mass weight (d.m.w.) of sediment, whilst TC was found at a concentration of 13.8 ng/g d.m.w. in sediment samples collected near the above-mentioned WWTPs (Siedlewicz et al. 2014).

Oxytetracycline is also characterised by being stable in low temperature areas when present in seawater and in sediments (Pouliquen et al. 2007; Li and Zhang 2016; Li et al. 2019). Its stability and lower bioavailability relate to the fact that the compound can bind with the divalent cations (e.g. Mg^2+^ and Ca^2+^) occurring in seawater (Zhang et al. 2015). Photoautotrophic microalgae, which are one of the primary producers in the aquatic ecosystem, are most sensitive to OTC. Also, OTC can reduce the growth rate of green algae (*Chlorella vulgaris*), or cyanobacteria (*Microcystis aeruginosa* and *Nodularia spumigena*) (Siedlewicz et al. 2020). Acute and chronic toxicity of OTC was already demonstrated using freshwater crustacean *Daphnia magna* (Wollenberger et al. 2000), zebrafish *Danio rerio* (Oliveira et al. 2013), and lake trout *Salvelinus namaycush* (Marking et al. 2011). In addition, the compound has been proven to act as endocrine disruptor. For example, in vitro exposure to OTC can lead to modulated gene expression and hormone production associated with steroidogenesis. In medaka fish *Oryzias latipes*, OTC and CTC at a concentration of 10 mg/L induced vitellogenin production in males, resulting in hormonal imbalance (Kim 2007). Other studies indicated that OTC may cause thyroid dysfunction and lead to reduced thyroid-stimulating hormone secretion in zebrafish (Yu et al. 2020). Banni et al. (2015) reported temperature-dependent effects of OTC at 1 and 100 μg/L on cellular and molecular parameters such as lysosomal membrane stability, antioxidant defence perturbations and heat shock response in the mussel *M. galloprovincialis* suggesting that risk from OTC exposure may be elevated at higher temperatures.

The blue mussels *Mytilus* spp. are keystone environmental engineers in various coastal ecosystems (Kochmann et al. 2008; Lauringson and Kotta 2016), including the brackish Baltic Sea. Apart from its ecological importance, this genus has also been of a commercial importance as it is widely cultured for food production. Mussels are also frequently used in pollution monitoring programmes (Bricker et al. 2014) as they are long-living sessile benthic species. By filtering large amounts of water, suspended organic matter and microalgae (Riisgård et al. 2013) they are exposed to various biologically active compounds which makes them good non-target model organisms to investigate ecotoxicological effects of pharmaceutical pollution. Yet, the knowledge about how OTC affects this key species is limited. Based on the studies of Banni et al. (2015), OTC exposure did not affect catalase activity whilst it did affect glutathione-S-transferase in *M. galloprovincialis* gills. Hence, OTC exposure may induce oxidative stress response and activate cellular detoxification processes including Phase I (monooxygenases belonging to the cytochrome P450 family) and Phase II xenobiotic biotransformation enzymes (transferases e.g., GSTs, UGTs, SULTs) and multixenobiotic resistance pumps (Phase III) in the mussel populations. On the other hand, 10-day use of the antibiotic can cause depletion of the tetracycline-sensitive microbiome and inflammations (Bassis et al. 2014). In mussels, commonly used markers characterising immunocompetence include the activity of enzymes such as alkaline phosphatase (ALP), asparaginase (ASP) and phenoloxidase (PO) in haemolymph samples. ALP activity changes in response to various biotic and abiotic factors, and a decrease in its activity was suggested as one of the indicators of reduced immunocompetence in invertebrates (Mazorra et al. 2002). Gut microflora, and specifically bacteria present in mussels can be a potential source of ASP enzyme, and the enzyme is produced by, e.g., *Pseudomonas* sp. and *Bacillus* sp., both sensitive towards OTC treatment (Mazorra et al. 2002). Phenoloxidase (PO) controls a process leading to melanin formation (i.e. melanization) in the pro-PO activating system (Cerenius and Söderhäll 2004). It is also responsible for a non-specific response of the host immune system. Amongst other antibiotics, OTC can modulate gene expression and production of hormone related to steroidogenesis (Gracia et al. 2007).

The main aim of this study was to understand if and how OTC affects xenobiotic detoxification systems, oxidative stress response and development of inflammatory responses in a model species *Mytilus trossulus*. Our goal was to identify detoxification mechanisms induced by OTC exposure such as mRNA levels of Phase I and Phase II biotransformation enzymes (NADPH-CYP450 oxidoreductases, glutathione-S-transferase) and xenobiotic efflux pump (P-glycoprotein) in the digestive system and gills of model mussels. In marine bivalves these enzymes are known to participate in defence against a broad range of xenobiotics (Smital et al. 2003; Zanette et al. 2013; González-Fernández et al. 2017). Immunological effects of OTC exposure were assessed based on measuring gene expression involved in the inflammatory pathways, including major vault protein (MVP), nuclear factor kappa B and NF-κB activating kinase—the inhibitor of NF-κB kinase subunit α (IKK1). Finally, we assessed the general health status of mussels exposed to OTC and the effect of OTC on bivalves steroidogenesis by analysing mitochondrial aromatisation efficiency.

## Materials and methods

### Chemicals

Oxytetracycline dihydrate (CAS number: 6153–64-6) was purchased from Sigma-Aldrich. Methanol (HPLC grade) for elution was purchased from POCH. To perform HPLC, we used formic acid and acetonitrile purchased from VWR. To determine aromatisation efficiency, (1β-^3^H)-androstenedione was used, purchased from Perkin Elmer. Methylene chloride, NADPH, Tris–HCl buffer, active charcoal and scintillation liquid: Ultima Gold™ XR LSC Coctail were purchased from Sigma Aldrich. For histological evaluation, we used haematoxylin and eosin purchased from Sigma Aldrich and formaldehyde, xylene and paraffin wax which were purchased from Chempur.

### Animal collection and laboratory exposure to OTCs

A total of 250 individuals of blue mussel *M. trossulus* were collected on board of R/V Oceanograf on 21 January 2021 from a 10 m deep sampling station (54°40′N, 18°33′E) located in the Gulf of Gdańsk at a distance from point-sources of pollution. The average size of collected mussels was 40 ± 3.5 mm, representing the proximate age of 4–5 years (calculated based on counting winter growth rings on the shell) (Sukhotin and Flyachinskaya 2009). All individuals were sexually mature, and sexing and sexual maturity was checked based on the gonadal subsample analysed under the light microscope for the presence of germ cells. Afterward, mussels were transported to the aquaculture laboratories and acclimated for 7 days before the experiment start. Mussels were randomly assigned to the control and OTC exposure groups, and each exposure condition was replicated three times with 35 individuals placed in one tank. The 15 l aquaria were filled with artificial seawater of salinity 7 ± 0.3 PSU (typical for Baltic waters), and the water was changed before adding OTC. The experiment was carried out at 10 °C for 10 days with aquaria daily monitored and constantly aerated during that time. During acclimation and exposure periods, mussels were fed daily with a polyculture algae mixture (4 ml of algae suspension per aquarium) containing species typical for the southern Baltic Sea obtained from the culture Collection of the Baltic Algae (https://ccba.ug.edu.pl/pages/en/home.php). OTC, diluted in water, was added to the culture system once at the start of the experiment and the nominal concentration was established as 100 µg/L. There were no changes of medium to replace the concentration during the exposure period. To analyse total OTC concentration in experimental conditions, a 50 mL water sample was collected each day, frozen and stored at − 80 °C. On day 10, the bivalves were removed and further processed as described below. For an assessment of enzymatic activity levels, 15 individuals from the control (5 per replicate) and 15 from OTC exposed conditions (5 per replicate) were selected, opened, their soft tissues dissected, frozen in liquid nitrogen and finally stored at − 80 °C. From another 30 clams (15—control and 15 OTC—exposed), haemolymph was collected from the adductor muscle of each mussel using microliter glass syringes whilst mitochondria from the remaining soft tissue were isolated and stored frozen at − 80 °C. For genetic analyses, twelve mussels from the control (four per replicate) and twelve from the OTC positive conditions (four per replicate) were dissected and gills, digestive system and rest of the soft-tissue (gonads and muscles) were separately frozen in liquid nitrogen and stored in a freezer at − 80 °C. Finally, using the similar pattern as described above, nine individuals from the control and nine individuals from the OTC treated group (three per replicate in both cases) were separated, dissected and the soft tissue stored in Davidson fixative for histology. Mortality was checked daily and the overall survival (%) in both experimental conditions was calculated as the number of recovered alive individuals during sampling divided by the number of specimens inserted in the aquaria at the start, times 100.

### Oxytetracycline analysis in water

Collected water samples were thawed at room temperature (20 °C). Then 2 mg/mL of EDTA was added to each sample to release the OTC from its complexes with Mg^2+^ and Ca^2+^ ions. Next, solid phase extractions (SPE) was performed during which 50 mL of each sample was loaded onto STRATA-X Phenomenex® 33 μm Polymeric Reversed Phase (200 mg/3 mL) pre-conditioned with 3 mL methanol (LC–MS grade) and followed by the addition of 3 mL ultrapure water. The extracts were eluted with 3 mL methanol, dried in an N_2_ atmosphere, and suspended in 1 mL of ultrapure water. Tests with spiked saltwater highlighted min. Ninety-seven percent recovery of OTC during extraction.

**Fig. 1:**
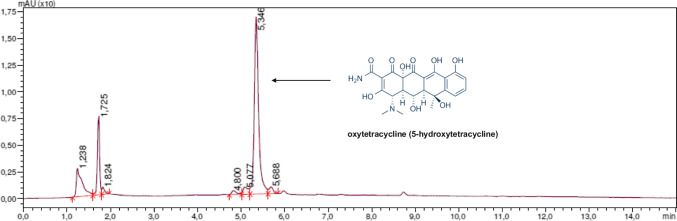
HPLC–DAD (356 nm) chromatogram of oxytetracycline in the extract of experimental water

The oxytetracycline standards and SPE-cleaned extracts were analysed with the high-pressure liquid chromatography (HPLC) method with diode-array detection (DAD) (Nexera, Shimadzu). The chromatographic separation was performed on a Gemini NX-C18 column (150 × 4.6 mm × 5 μm, Phenomenex®). The column and autosampler temperature were kept at 27 °C and 10 °C, respectively. Solvent A was composed of water with 0.1% formic acid (≥ 99%, VWR Chemicals, United Kingdom), and solvent B was acetonitrile (HPLC grade, POCH, Poland). The solvent gradient used for elution was as follows: start with 90% solvent A and a decrease in its concentration to 65% over 10 min, 2 min in isocratic elution of 65% solvent A, and finally back to the 90% solvent A over 3 min (totally 15 min of a single run). The flow rate of the mobile phase was 1.2 mL/min and the sample injection volume was 25 μL. The limit of OTC detection for this method was 0.039 μg/L (the signal-to-noise ratio of 3), and the limit of quantification was 0.078 μg/L (the signal-to-noise ratio of 10). The accuracy (ration of found to nominal concentration) and precision (relative standard deviation of analysis) was 95.9–102.2% (mean 97%) and 0.1–3.9% (mean 3.1%), respectively. The analytical wavelength was 356 nm. The retention time of OTC was 5.25–5.35 min. An exemplary chromatogram of HPLC–DAD analysis of OTC in extracts is presented in Fig. 1.

### Biochemical analyses

Enzymatic activities were measured using a UV–VIS Spectrophotometer, Beckman Coulter, Synergy 2 Multi-Mode Reader (BioTek), and Beckman LS 6000 IC counter. In haemolymph samples, activities of alkaline phosphatase (ALP), asparaginase (ASP) and phenoloxidase (PO) were measured according to methods described in Supplementary Material S1a. Oxidative stress biomarkers were measured in whole soft tissue of each individual. Individual samples were homogenised manually with a Teflon-pestle homogeniser in ice-cold buffer containing 50 mM Tris-H_2_SO_4_, pH 7.6 with 0.1 mM EDTA, 1 mM PMSF, 2 mM DTT and 0.2% Triton X-100. The homogenates were centrifuged at 14,000 RCF for 30 min at 4 °C. The supernatants were transferred into fresh tubes and used for analysis. Cytosolic protein was determined by the Lowry method (1951) with the modification of Peterson (1977). Enzyme activities of glutathione S-transferase (GST) and catalase (CAT) were measured, according to methods described in Supplementary Material S1a. Total glutathione concentration (tGSH), total antioxidant capacity (TAC), malondialdehyde (MDA) and carbonyl (CBO) levels were measured spectrophotometrically based on the method described in the Supplementary Material S1b. In mitochondria isolated from whole soft tissue of each individual, the aromatisation efficiency (AE) was quantified. The method of mitochondrial isolation and measurement of AE were included in Supplementary Material S1c.

### Quantitative RT-PCR

Total RNA was extracted from the digestive gland and gills of control and OTC-exposed mussels using Absolutely RNA Miniprep Kit (Agilent Technologies, Santa Clara, CA, USA) according to the manufacturer’s protocol. The purified RNA was eluted in a 30 μL Elution Buffer. The purity and concentration of the RNA were evaluated by NanoDrop 2000 Spectrophotometer (Thermo Fisher Scientific, Waltham, MA, USA). Samples with a criterion of A260/280 ≥ 2.00 were stored at − 80 °C until further analysis. cDNA was obtained from 1 μg of total RNA using SensiFAST cDNA Synthesis Kit (Meridian Bioscience Inc., Cincinnati, OH, USA) according to the manufacturer’s instructions. Quantitative PCR was carried out using CFX Connect Real-Time PCR System (Bio-Rad, Hercules, CA, USA) and SensiFAST No-Rox Kit (Meridian Bioscience Inc., Cincinnati, OH, USA). Expressions of target genes were calculated based on the delta-delta Ct method. A combination of housekeeping genes (GAPDH and COX1) was used for normalisation. Reference (housekeeping) genes were selected using the BestKeeper software. The primers’ sequences are presented in Table 1. Amplification of specific transcripts was confirmed based on melting curve profiles.

**Table 1:** Primer sequences for the housekeeping and target genes

Gene	Forward primer (3′-5′)	Reverse primer (3′-5′)	NCBI accession number
CYP1L1	GTCCGTGGGGCTTTCCTATT	CGGTGCATTTTCCAACGAGG	JX885878.1
COX1	GGAAGTCGAGTCGTGGTGAC	GCTCCAGGATGCCCTAACTG	KF220398.1
GAPDH	CTGAGGGTCCAATGAAGGGTG	CCTGTTGTCTCCACGGAAATC	KJ808669.1
GSTϛ3	TTTGCTGCGGCTGGTAAAGA	AAATGGCACCACTCTGGTTGA	JX485638.1
NF-κB	TCTACATGCAACCCCAAGGC	GGAGTAAGTTGTGACCCCCG	KF051275.1
IKK1	GTGGTGCTAGTCCTGATCCA	GCTTCACCCCATGCTTTCTT	KF015301.1
MVP	GGAAAAGAGAACAGCTGGGGA	CCCTAGCAACACCTTGTCTGT	AF172605.1
HSP70	ATACCCACTTGGGTGGTGAAG	GACAGCACGCTTGTTTTCACT	AF172607. 1
PGY1	ACACACTAGTTGGAGAGCGTG	CAAAGCTCTGGCAATGGCTAC	AF159717.1

### Tissue preservation and histological examination

Whole tissues from certain individuals were removed from their shells and preserved in Davidson fixative containing glacial acetic acid, 37–40% formaldehyde, 96% ethanol and isotonic sodium chloride. After 3 days, the samples were moved to a 10% buffered formaldehyde solution. Next, samples were dehydrated through an ascending ethanol series, cleared in xylene, using a tissue processor CITADEL 2000 (Thermo Fisher), and embedded in paraffin wax. Blocks containing mussel tissues were cut into 3 μm thick sections and prepared sections were moved onto microscopic slides. Next, tissue was cleared in xylene, rehydrated in descending ethanol concentration bathes, soaked with tap water and finally stained with haematoxylin and eosin (standard H&E staining protocol). Prepared microscopic slides were screened for the presence of histopathologies under the light microscope Nicon Eclipse. Sex ratio (SR) was calculated as females to males ratio whilst gonadal index (GI) was assessed and calculated according to Wenne and Styczyńska-Jurewicz (1985). Scoring of histopathologies was based on their presence and/or severity (no lesion marked as 0 whilst its presence as 1). Occurring lesions were pooled by tissue type (gills, gonads and digestive system).

### Statistics

Data normality was tested using Shapiro–Wilk test. Statistical significance of differences between groups was verified with the Student t-test for data with normal distribution or Mann–Whitney *U* test for data with non-normal distribution. The differences were considered significant at *p* < 0.05. The results are presented as means ± standard errors of the mean (S.E.M). Calculations were performed using the STATISTICA 13 software (Statsoft, Kraków, Poland), and figures were prepared in the Sigma-Plot 11 software.

## Results

### Stability of oxytetracycline in water

Medium OTC concentrations measured in experimental tanks were 100.35 ± 4.53 μg/L after 24 h, 99.58 ± 13.47 μg/L after 48 h, 94.92 ± 9.34 μg/L after 120 h and 87.81 ± 16.89 μg/L after 240 h (values show means ± S.E.M.).

### Mussel survival rate

In the control group, the survival rate was approximately 90% (eleven dead individuals within 10 days of the experiment (three, four and four—respectively in each repetition) per group of 105 bivalves introduced into the control group, whilst in the OTC exposed group the survival rate was approximately 70% (31 dead individuals within 10 days of experiment (ten, twelve and nine—respectively in each repetition) per group of 105 bivalves introduced into the OTC-treated group. The surviving individuals from the OTC exposed group and the similar equivalent of those from the control were used for biochemical, histological and genetic analyses.

### Enzymatic activities

The activities of three enzymes: alkaline phosphatase (ALP), asparaginase (ASP) and phenoloxidase (PO) were determined in the haemolymph of *M. trossulus* (Table 2). In the OTC-treated group, ALP activity in the haemolymph was 189.30 ± 20.46 U/L and was not different from the activity determined in the control group (185.51 ± 11.60 U/L). Likewise, no change in ASP activity was observed in the haemolymph of exposed bivalves (825.64 ± 116.95 U/L in the control group and 862.66 ± 52.17 U/L in the OTC treated animals). However, in the haemolymph of individuals treated with OTC, a higher PO activity was determined as compared to PO activity measured in the haemolymph of the control group (30.95 ± 3.33 U/L and 17.95 ± 2.75 U/L, respectively). Activities of antioxidative enzymes and the content of low molecular antioxidants measured in the remaining mussel tissues are presented in Table 2. GSTs activity in the control group oscillated around 57.61 ± 3.12 nmol/min/mg protein whilst in the OTC-treated group GSTs activity reached 52.81 ± 2.79 nmol/min/mg protein, highlighting no significant differences between both tested groups. CAT activity measured in OTC-exposed mussels was 12.14 ± 1.73 U/mg protein, whilst the result in the control group was 10.67 ± 1.83 U/mg protein. The concentration of tGSH measured in bivalves from the control group was 16.46 ± 0.91 nmol/mg protein whilst in those exposed to OTC, we measured 16.68 ± 0.84 nmol/mg protein. Similarly, the TAC level in mussels from the control group was 686.13 ± 14.80 nmol/mg protein whilst in the OTC-exposed group, we measured 735.44 ± 19.95 nmol/mg protein. Thus, there were no statistically significant differences in the amount of low molecular antioxidants between the two mussel groups. The amount of MDA in both groups of mussels remained at the same level; 2.69 ± 0.002 nmol/mg protein in the control and 2.66 ± 0.01 nmol/mg protein in the OTC-treated ones. Also, no significant difference in the amount of formed protein peroxidation products between control animals (3.04 ± 0.32 nmol/mg protein) and OTC-treated animals (4.72 ± 1.18 nmol/mg protein) was found. The efficiency of the androgen aromatization process differed between mussels exposed to OTC and those from the control (Table 2); however, the difference was not statistically significant (*p* = 0.35). Yet, a decreasing trend in AE in blue mussels exposed to OTC (19.67 ± 3.58 pmol/h/mg protein) when compared to those from the control (24.34 ± 2.16 pmol/h/mg protein) was noted.

**Table 2:** OTC effect on biochemical parameters in exposed blue mussels (ANOVA Mann–Whitney *U* test). Data presented as mean ± S.E.M. Abbreviations: *OTC*, oxytetracycline; *ALP*, alkaline phosphatase; *ASP*, asparaginase; *PO*, phenolooxidase; *GST*, glutathione S-transferase; *CAT*, catalase; *tGSH*, total glutathione (GSH + GSSG); *TAC*, total antioxidant capacity; *CBO*, carbonyls; *MDA*, malondialdehyde; *AE*, aromatisation efficiency. Asterisk (*) indicates significant effects

	Mean ± S.E.M		*p*-value
	Control group	OTC- treated group	
Haemolymph enzymes activities (U/L)			
ALP	185.51 ± 11.60 *n* = 10	189.30 ± 20.46 *n* = 10	*p* = 0.63
ASP	825.64 ± 116.95 *n* = 12	862.66 ± 52.17 *n* = 12	*P* = 0.291
PO	17.95 ± 2.75 *n* = 14	30.95 ± 3.33 *n* = 14	*p* = 0.006*
Oxidative stress biomarkers			
GST (nmol/min/mg protein)	57.61 ± 3.12 *n* = 14	52.81 ± 2.79 *n* = 14	*p* = 0.158
CAT (U/mg protein)	10.67 ± 1.83 *n* = 12	12.14 ± 1.73 *n* = 12	*p* = 0.503
tGSH (nmol/mg protein)	16.46 ± 0.91 *n* = 14	16.68 ± 0.84 *n* = 15	*p* = 0.914
TAC (nmol/mg protein)	686.13 ± 14.80 *n* = 14	735.44 ± 19.95 *n* = 15	*p* = 0.145
CBO (nmol/mg protein)	3.04 ± 0.32 *n* = 14	4.72 ± 1.18 *n* = 15	*p* = 0.59
MDA (nmol/mg protein)	2.69 ± 0.002 *n* = 14	2.66 ± 0.10 *n* = 15	*p* = 0.59
Mitochondrial aromatisation efficiency			
AE (pmol/h/mg protein)	24.34 ± 2.16 *n* = 7	19.67 ± 3.58 *n* = 7	*p* = 0.386

### mRNA expression of biotransformation enzymes and inflammatory response genes

**Fig. 2:**
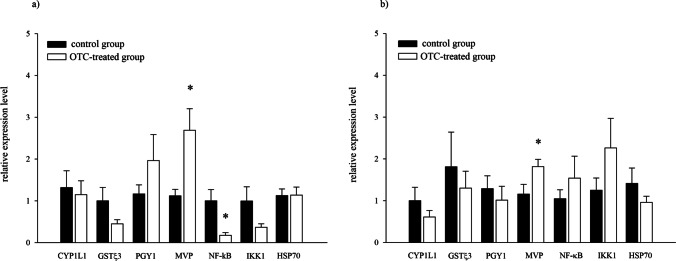
Effect of OTC exposure on the selected genes expression in a) digestive system (*n* = 10; both groups) and b) gills (*n* = 9; both groups) of *M. trossulus*. mRNA of target genes: CYP1L1—cytochrome P450 family 1-like 1 protein, GSTϛ3—glutathione S-transferase sigma 3, PGY1—p-glycoprotein, MVP—major vault protein, NF-κB—nuclear factor kappa B-a, IKK1—inhibitor of nuclear factor kappa-B kinase-1, HSP70— heat shock protein relative to housekeeping genes (COX1—cytochrome c oxidase subunit 1 and GAPDH—glyceraldehyde-3-phosphate dehydrogenase). Asterisks indicate the values that are significantly different between the control and OTC-exposed group in the same tissue (*p* < 0.05)

The expression of genes encoding proteins involved in the detoxification and inflammatory processes was measured in the digestive system (DS) and gills of OTC-treated and control mussels. No differences in CYP1L1 gene expression were found in both experimental conditions, neither in the digestive system nor the gills (Fig. 2a, b). Likewise, there were no differences in the expression of the detoxification phase II gene encoding GSTϛ3, in both groups and tissue types the gene expression was maintained at similar levels (Fig. 2a, b). When analysing the expression of genes encoding proteins in the phase III of detoxification, there were no differences in PGY1 protein expression between OTC-treated animals and controls, neither in DS nor in gills (Fig. 2a, b). In contrast, a 2.4 times higher expression of the MVP gene in the DS tissues of the OTC-treated bivalves group (*p* = 0.009) was found (Fig. 2a). Also, a statistically significant increase in MVP gene expression (1.5-fold) was determined in the gill tissues in OTC group (*p* = 0.027) (Fig. 2b). There were no tissue-related and exposure-related differences in the expression of HSP70 gene (Fig. 2a, b). Yet, a 3.4-fold decrease in the expression of the NF-κB gene, an inflammatory marker, in the DS of mussels in the OTC-exposed group (*p* = 0.024) was noted (Fig. 2a). No such decrease was noted in the gills (Fig. 2b). At the same time, no tissue-related or OTC-related changes in the expression of the kinase controlling NFKB – IKK1 were observed (Fig. 2a, b).

### Histological representation of tissue pathologies

**Fig. 3:**
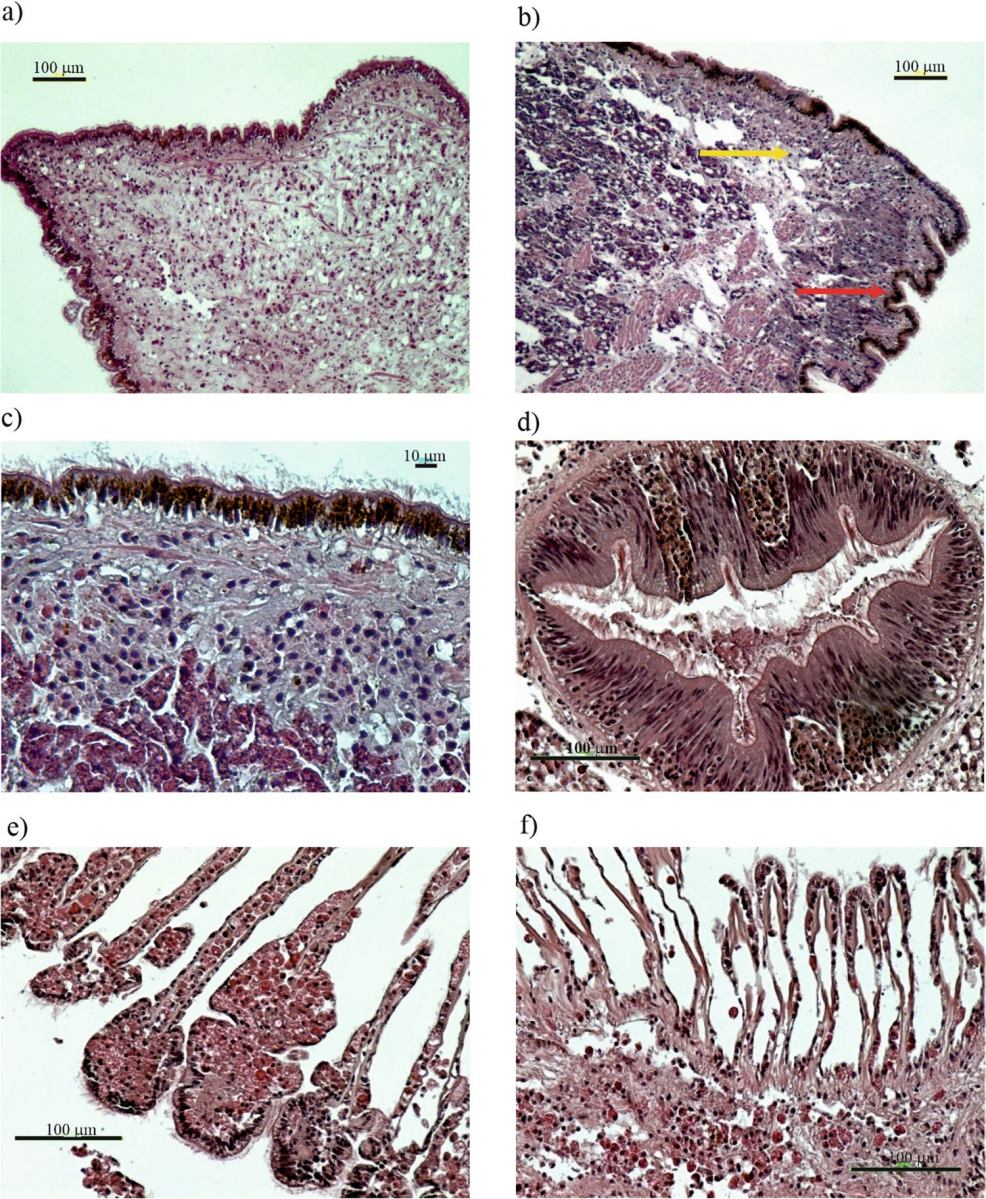

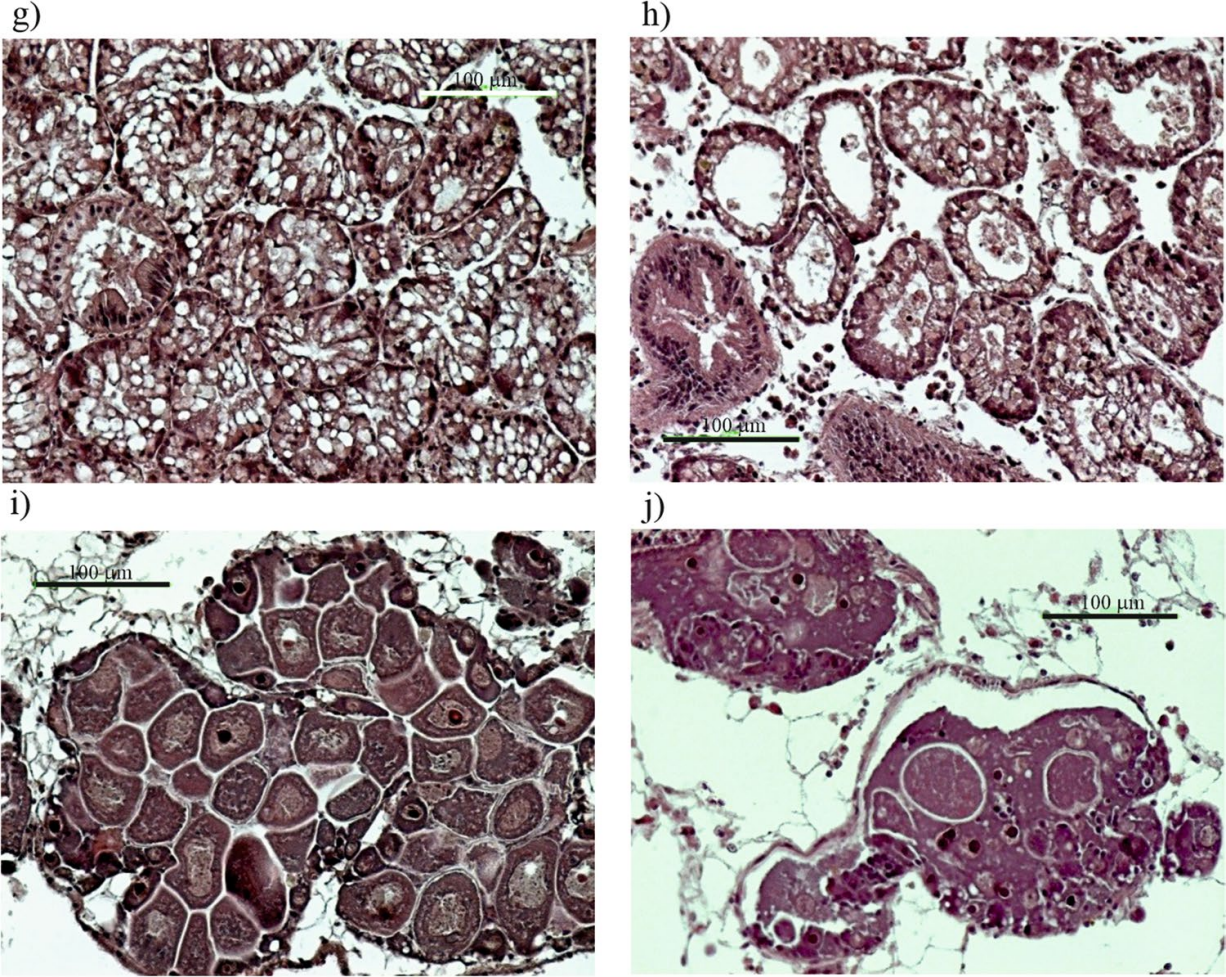
Cross-sections through *M. trossulus* tissues: normal appearance of mantle tissues in non OTC-treated individual (**a**) and mantle alterations in OTC-treated mussel with ‘brown cells’ (red arrow) and bacterial colonies (yellow arrow) (**b**); higher magnification of brown cells found in mantle epithelium (**c**) and in the upper part of the digestion system of the same OTC-treated mussels (**d**); swollen gill filaments of bivalves from the OTC untreated group (**e**) and gill filaments of an OTC-treated individual with increased infiltration of haemocytes and granulocytes (**f**); vacuolised structure of otherwise normal structure of digestive tubules in an individual from the control (**g**) and advanced atresia of the digestive follicles in the OTC-treated individual (**h**), female OTC-non treated follicle with normal mature oocytes (**i**) and abnormal gonadal tissue with atresia in OTC-treated female tissue (**j**)

The sex ratio showed the dominance of females in both experimental conditions (SR = 2 in the control and SR = 1.25 in the OTC-treated mussels). No statistically significant difference in the gonadal development between mussels from the control and OTC-treated ones [gonadal index (GI) = 2.77 and GI = 2.66, respectively] was found. A section through the normal mantle tissue of the control mussel (Fig. 3a) showed connective tissue along with muscle fibres, all surrounded by well-differentiated epithelium with small cilia, within which cuboidal secretory cells were visible. In a section through the mantle tissue of an individual after OTC exposure (Fig. 3b), connective tissue along with muscle fibres was present. In the epithelium of the mantle and DS, yellowish-brown objects were clearly visible (Fig. 3c, d). These may be either lipofuscin or melanin pigmentation, so-called brown cells. Pigmented cells of that type were seen both in control (five individuals) and in OTC-treated individuals (seven individuals). Such cells are often associated with ongoing degradation processes. Phagocytes and necrotic areas were also visible in the mantle cavity. In one OTC-exposed individual, an aggregation of different haemocyte types forming granulocytoma surrounded by highly degraded secretory or glandular mantle cells was seen. Figure 3e shows a cross section through the gill filaments of bivalves from the OTC untreated group with the terminal part of the filament infiltrated with haemocytes and clearly swollen. Figure 3f shows a cross-section through the gill filaments of an OTC-treated individual. Increased infiltration of haemocytes and granulocytes along with disruption of the tissue structure was observed within the basal tissue. The conditions described above (Fig. 3e, f) occurred in both control and OTC-treated mussels. However, they were more often observed in the OTC-treated group (3 and 5 individuals, respectively). The normal structure of the mussels’ hepatopancreas from the control group is shown in Fig. 3g. Advanced atrophy of the digestive follicles along with vacuolization of the absorptive cells was observed in the OTC-treated individual (Fig. 3h). Atrophic follicles had enlarged lumen indicative of different stages of follicle wall degradation with often co-occurring severe vacuolisation of the digestive tubules. Changes in the digestive tubules were more often present in OTC-treated individuals (seven individuals) when compared to control (three individuals). The normal appearance of female gonads with mature oocytes is presented in Fig. 3i. Gonadal atresia (lysis of gonadal material), a regressive change occurring in the female gonads of an OTC-treated individual, is shown in Fig. 3j. This change was only present in OTC-treated mussels (four out of five females affected). The detailed histological characteristic of mussels is presented in the supplementary material (Tab. S1).

## Discussion

This study presents results describing the effect of OTC on xenobiotic detoxification systems, oxidative stress response, general health and development of inflammatory responses in a non-target species *M. trossulus*. Our hypothesis was that the exposure to OTC will result in changes of mRNA levels of Phase I and Phase II biotransformation enzymes (NADPH-CYP450 oxidoreductases, glutathione-S-transferases) and xenobiotic efflux pump (P-glycoprotein) in the digestive system and gills of mussels. Our stated hypothesis has not been confirmed. Measuring gene expression involved in the inflammatory pathways; major vault protein (MVP), nuclear factor kappa B and NF-κB activating kinase—the inhibitor of NF-κB kinase subunit α (IKK1), allowed for assessing potential immunotoxicity of OTC to a model species. Our results highlighted induction of an inflammatory reaction and the absence of detectable signs of oxidative stress in mussels exposed for 10 days to OTC at a concentration of 100 µg/L. However, experiments using marine invertebrates provided evidence that OTC induces oxidative stress in those organisms. Experimental exposure of a crab *Portunus trituberculatus* larvae to low doses of OTC (0.3, 3 and 30 µg/L for 3 days) suppressed the antioxidant system of these animals which was demonstrated as the decrease of GSTs and SOD activity and subsequent increase of lipid peroxidation products (Ren et al. 2017). In another study, a freshwater snail *Lymnaea stagnalis* was exposed to 200 ng/L of OTC for 3 days. The effect of OTC was revealed by increased expression of the CAT gene and decreased expression of the gene encoding HSP70 (Gust et al. 2013). Exposure studies using OTC have also been conducted on marine bivalves, although not extensively. For example, Banni et al. (2015) reported a decrease in lysosomal membrane stability and an increase in MDA accumulation as well as a significant increase in CAT and GSTs transcript levels in *M. galloprovincialis* exposed to 100 µg/L OTC. The antioxidant chaperone and stress marker such as HSP27 mRNA were also significantly upregulated in OTC-treated animals. In Buratti et al. (Buratti et al. 2010), *M. galloprovincialis* was exposed to a wide range of OTC concentrations (from 0.1 to 10 µg OTC/L) for 4 days. 1 μg/L OTC-treated individuals showed an increase in the inducible HSP70 protein expression, with a maximum effect of about 185% vs control values (Buratti et al. 2010). Given the available literature studying OTC exposure effects on marine species and the extent of reported cellular and molecular responses, our results clearly indicate that *M. trossulus* mussels showed no signs of oxidative stress after 10 days of exposure to 100 µg/L OTC. We observed no differences in the expression of the gene encoding one of the numerous heat shock-protein family members, the HSP70 protein, neither in the digestive system nor in the gills of OTC-treated mussels vs the control group. Apart from the exposed species (*M. trossulus* here and *M. galloprovincialis* in the above mentioned works), the main differences between our experiment and the work of Buratti et al. (Buratti et al. 2010) and Banni et al. (2015), are longer exposure of the Baltic blue mussels (10 days versus 4 days) at an identical OTC dose (100 µg/L), lower exposure temperature (10 °C versus 16° C) and different salinity. These abiotic factors are most likely enough for the results of both works to be different, despite the same concentration of OTC. The main factor affecting the expression of heat shock proteins is an increase in water temperature. When exposed to acute heat stress, both mussel species showed increased levels of the molecular chaperones HSP70 and HSP90 (Tomanek and Zuzow 2010). Also, thermal dependencies of gene expression may partially explain such differences in the obtained results as we compare two mussel species having different thermal optima.

In the OTC-exposed mussels, a significant increase in the expression of the gene encoding MVP was observed in both the digestive system and the gills. The major vault protein is the predominant component of the largest ribonucleoprotein particle present in eukaryotic cells. MVP vaults have been associated with multidrug resistance, nuclear–cytoplasmic transport, autophagy, signal transduction pathways and innate immunity (Berger et al. 2009). Although most of the literature on MVP protein focuses on mammalian cells, a group of MVPs has been identified in several marine organisms such as catfish (as a protein involved in cancer progression) (Margiotta et al. 2017), sea urchin (an important factor during embryonic development; Stewart et al. 2005), and in bivalves (participating in the phase III system of detoxification; Luedeking and Koehler 2004). An increase in MVP expression is observed in humans in the innate response during both viral and bacterial infection. For example, a high induction of MVP expression in human cells in response to infection with Epstein-Barr virus (EBV) has been reported (Mrázek et al. 2007). Likewise, the results of Liu et al. (2012) demonstrated that MVP induces type I IFN production upon hepatitis C virus infection in humans. MVP protein is also involved in the immune response in bacterial infections. It was shown that MVP proteins are essential for host resistance against lung infection in humans by *Pseudomonas aeruginosa* based on the effective internalisation and clearance of the pathogen (Kowalski et al. 2007). The molecular mechanisms underlying the role of MVP in this process are unclear, but the authors suggest that either cytoskeletal regulatory functions necessary for lipid microdomains formation or impacts on signal pathways like the MAPK might be involved. Infection caused by *Listeria monocytogenes* has also been shown to increase MVP expression, that in turn masks intracytosolic pathogens from autophagic recognition and clearance (Dortet et al. 2011). Our results indicate a significant increase in MVP expression in OTC-exposed mussel tissues. This may present an immune response to the presence of an infectious agent—most likely of bacterial origin—which was confirmed by increased PO activity in the haemolymph of OTC-treated bivalves (Tab. 2). PO is a key enzyme in the melanisation cascade that also participates in the innate immune response against microbial infections in invertebrates (Cerenius and Söderhäll 2004). The enzyme, usually synthesised as an inactive zymogen pro-PO, is activated to its counterpart PO via proteolytic cleavage of the prophenoloxidase-activating system (PPAE) starting cascade reaction resulting in melanin synthesis as a response to the pathogens surface molecules detection (Cerenius et al. 2010). Intracellular PO activities have been reported in several bivalve species, including *Saccostrea glomerata*, *Pinctada imbricata*, *Ruditapes philippinarum*, *Chamelea gallina* and *Tapes decussatus* (Allam and Raftos 2015) and blue mussels *M. edulis* (Pipe and Coles 1995; Zentz et al. 2002). Likewise, activation of the PPAE system has been observed in shrimps *Penaeus vannamei* infected with *Vibrio parahaemolyticus* (Boonchuen et al. 2021) and in crab *Scylla paramamosain* infected with *V. alginolyticus* (Yang et al. 2014). Our results indicate that the OTC-treated mussels were characterised by the presence of regressive changes in the female gonads and infection with tetracycline non-sensitive bacteria after 10 days of antibiotic treatment. Our results also highlighted activation of the PPAE system and increased PO activity in the haemolymph as well as brown pigmentation accumulation in tissues, mainly in the mantle epithelial cells. Oocyte atresia was observed in female gonads in the OTC group of mussels. Necrotic oocytes (exhibiting severely disrupted cellular membrane) were also recorded in atretic gonadal follicles. Observed gonadal atresia may eventually lead to malfunction of the gametogenesis process and therefore to partial or total temporal sterilisation of an affected individual. Gonadal atresia has also been shown to be associated with the presence of pollution and endocrine-disrupting chemicals (EDc) (Kronberg et al. 2021). Oxytetracycline may be considered an EDs factor, but its endocrine influence was observed only at milligramme concentrations (Gracia et al. 2007; Kim 2007). In our study, OTC at 100 µg/L admittedly induced a decrease in tissues AE, although it was not statistically significant. The reduced AE may be also related to alterations found in the gonads, as our previous results highlighted that AE is most effective in the well-developed female gonads (Hallmann et al. 2019).

Mantle tissue observations resulted in the detection of various lesions in OTC treated individuals, yet some of these lesions were also observed in individuals from the control. In addition to melanin and lipofuscin accumulation, clusters of immune cells and necrotic changes were observed in OTC-treated individuals. When immunocytes aggregate in a characteristic manner to form small and large clusters in both interstitial tissue and haemolymph, it is termed a nodular-type inflammatory response (Galloway and Depledge 2001). In invertebrates, nodule formation is normally thought to be a consequence of immunocyte aggregation following bacterial challenge (Rowley 1996). Granulocyte infiltration has also been noticed in the mantle tissue of OTC mussels group. This supports the granuloma-like inflammatory response hypothesis (de Vico and Carella 2012). In bivalves, gills are amongst the main target organs for pollutant accumulation due to their primary roles in ingestion and respiration, and due to their close contact with ambient water. Therefore, the gill epithelium is highly exposed/prone to both viral and bacterial infections. Haemocytes move to sites of infection (inflammatory sites), which is the first step in phagocytosis. Bivalve haemocytes display positive chemoattraction towards products released by infectious agents ranging from multiple parasites over various bacterial species (Cheng and Howland 1979) to bacterial products such as lipopolysaccharides (Howland and Cheng 1982). The gill tissue of OTC-treated bivalves had a disrupted structure and a strongly marked infiltration of haemocytes, which again may confirm bacterial involvement in the inflammatory process. Inflammatory changes with haemocyte infiltration and granulocyte accumulation were also seen in the hepatopancreatic tissue of the OTC-treated mussels (2 cases). Additionally, advanced atrophy and vacuolisation of the digestive tubules were noted. This histopathological feature appears as a reduction in the thickness of epithelia accompanied by the enlargement of the digestive tubule lumen (Fig. 3f). Similar inflammatory changes in hepatopancreas tissue were observed in *M. galloprovincialis* from several sites of Bay of Biscay (Cuevas et al. 2015) and were related to the presence of parasites in the bivalve tissues and to a significant load of sediment pollution.

Ten-day exposure of mussels to oxytetracycline did not change the expression of genes encoding proteins involved in the detoxification processes. No effects on the expression of CYP1L1 or GSTϛ3 genes were observed; neither in the digestive system nor in the gills. Also, the expression of the gene encoding P-glycoprotein (PGY1) was not altered. In bivalves, the transcriptional inducibility of CYP450 monooxygenases by xenobiotics depends both on the xenobiotic and on the specific CYP450 isoform (Cubero-Leon et al. 2012; Zanette et al. 2013; González-Fernández et al. 2017). Despite this variability of response at the mRNA level, enzymatic activity of CYP450 monooxygenases is commonly elevated in *Mytilidae* family exposed to xenobiotics, possibly through post-transcriptional mechanisms (Bebianno et al. 2007; Counihan 2018; Falfushynska et al. 2019). The lack of induction of Phase I enzyme activities in OTC-exposed mussels suggests that the CYP1L1 pathway is not involved in OTC metabolism in the main organs participating in xenobiotic uptake (the gills) and biotransformation (the digestive gland) of *M. trossulus*. GSTs plays an important role in detoxification of xenobiotics by conjugating them to glutathione, thereby making them less toxic and more easily excretable (Sheehan et al. 2001; Allocati et al. 2018). GSTs commonly act on xenobiotics modified by Phase I biotransformation enzymes such as CYP450 monoxygenases, but some xenobiotics may bypass Phase I biotransformation and are directly detoxified by GSTs (Allocati et al. 2018). Our results differ from the observations of Banni et al. (2015), where *M. galloprovincialis* bivalves exposed to 1 µg OTC/L and 100 µg OTC/L presented both an increase in expression of the gene encoding CAT and GSTs. The authors of this report clearly indicate that oxidative stress was present in mussel tissues expressed by decreased lysosomal membrane stability and by increased lipid peroxidation products. These results correlate with an increase in the expression of the gene encoding HSP70. Our results also showed inhibition of the expression of the gene encoding the nuclear factor-κB (NF-κB) in the digestive system of OTC-treated mussels. Transcription factors of the NF-κB family play a pivotal role in the inflammatory and immune response (Ghosh et al. 1998). The inactive NF-κB complex is activated in response to a variety of stimuli, including viral and bacterial infection, exposure to proinflammatory cytokines, mitogens and growth factors and stress-inducing agents (Karin and Ben-Neriah 2000). The subcellular location of NF-κB is controlled by a family of inhibitory proteins, IκBs and the IκB kinase (IKK) play a major role in NF-κB activation. No changes in IKK1 kinase expression were found in OTC-treated mussel tissues, suggesting that it had no inhibitory effect on the NF- κB gene expression. Ben et al. (2019) reported that increased MVP expression is responsible for the inhibitory effect of NF- κB pathway signalling. This process involves macrophages and is responsible for reducing inflammation in metabolic diseases in mammals. MVP interacts with TNF receptor-associated factor 6 (TRAF6) preventing its recruitment to interleukin 1 receptor associated kinase 1 (IRAK1) and subsequent oligomerization and ubiquitination. Overexpression of MVP and its α-helical domain inhibits the activity of TRAF6 and suppresses macrophage inflammation (Ben et al. 2019). A similar correlation between upregulation of MVP gene expression and downregulation of NF-κB gene in the digestive system of OTC-treated mussels was observed in our study. Hence, the observed inflammatory reaction is suppressed by MVP protein-mediated inhibition of the NF- κB pathway in OTC-exposed mussels. This mode of regulation is unknown in bivalves and requires further study.

Elevated mortality in OTC-exposed mussel groups in comparison to control may be attributed to the observed inflammatory process of undefined origin. Yet, symbiotic bacteria in molluscs are involved in many physiological processes, assisting their hosts in digestion, assimilation of nutrients, enhancing immunity and protecting against pathogens (Nicolai et al. 2015). Some bacteria can fix nitrogen or supply steroids and vitamins (Rowland et al. 2018). The activities of bacterial enzymes such as cellulase or chitinase were demonstrated in the digestive systems of molluscs (Pinheiro et al. 2015). Studies on the microbiome of *Mytilus* sp. show that mussels live in symbiosis with many bacterial species belonging to genera such as *Acinetobacter*, *Photobacterium*, *Moraxella*, *Aeromonas*, *Micrococcus* and *Bacillus* (Cavallo et al. 2009), some of which may be pathogenic: *Psychrilyobacter*, *Brochothrix*, *Alteromonas*, *Lutibacter*, *Donghicola*, *Vibrio*, *Pseudomonas* or *Mycoplasma* (Griffin et al. 2021). Besides, the mussel gut microbiome is highly diverse during summer and autumn, which is attributed to the increased physiological activity of these bivalves. In winter, there is a noticeable decrease in the microbiome (Pierce and Ward 2018). That is also related to the reduced metabolic activity of these animals. Ecologically, a heterogeneous microbiome is important to the host and can suppress the colonisation of pathogens such as *Vibrio*, *Pseudomonas* and *Aeromonas—*bacteria that frequently attack mussels and that are responsible for their increased mortality (Kwan and Bolch 2015). We confirmed the presence of bacteria in bivalve tissues indirectly by measuring asparaginase (ASP) activity in their haemolymph. Microorganisms are known to produce this enzyme including *Escherichia coli*, (Ghasemi et al. 2008), *Vibrio* (Kafkewitz and Goodman 1974), *Aerobacter*, *Bacillus*, *Serratia*, *Xanthomonas*, *Photobacterium* (Peterson and Ciegler 1969), *Streptomyces* (DeJong 1972), *Pseudomonas aeruginosa* (El-Bessoumy et al. 2004) and *Aspergillus tamari* (Sarquis et al. 2004).

## Conclusions

Ten-day exposure of the blue mussel *M. trossulus* to oxytetracycline at a concentration of 100 µg/L did not provoke cellular oxidative stress and did not affect expression levels of the genes involved in detoxification processes in these bivalves. Mussels were suffering from inflammations of undefined origin and higher frequency of regressive changes, but we also detected increased PO activity in the haemolymph and an activation of the gene expression encoding MVP.

## Supplementary Information

Below is the link to the electronic supplementary material.

Supplementary file1 (DOCX 14 KB)

Supplementary file2 (XLSX 20 KB)

Supplementary file3 (DOCX 68 KB)

## Data Availability

Raw data and all other material will be available upon request.
