# Supplementary material for: Oxytetracycline-induced inflammatory process without oxidative stress in blue mussels Mytilus trossulus
Source: Environ Sci Pollut Res Int. 2023 Jun 10;30(33):80462–77. doi: 10.1007/s11356-023-28057-z (PMC10345040; doi:10.1007/s11356-023-28057-z)
Supplement: Supplementary file 1 — Supplementary file1 (DOCX 14 KB) [file 11356_2023_28057_MOESM1_ESM.docx]

Table S1. Histological characteristic of analysed mussels. IG – gonadal index, IR – inflammatory reaction, vacuolis. – vacuolisation, BC – brown cells, NC – necrotic cells

| **Exposure** | **No.** | **Gonads** | | | | **Gills** | | **Digestive system** | | **BC** | **NC** | **Other** |
| --- | --- | --- | --- | --- | --- | --- | --- | --- | --- | --- | --- | --- |
|  |  | sex | IG | atresia | IR | IR | Oedema | IR | Atrophy / vacuolis. | epithelium | epithelium |  |
| **Control1** | 1 | ♀ | 2 | 0 | 0 | 1 | 0 | 0 | 0/1 | 1 | 0 | 0 |
|  | 2 | ♂ | 3 | 0 | 0 | 0 | 0 | 0 | 0/0 | 0 | 0 | 0 |
|  | 3 | ♀ | 4 | 0 | 0 | 0 | 0 | 0 | 0/0 | 1 | 1 (mantle/foot) | 0 |
| **Control2** | 1 | ♀ | 3 | 0 | 0 | 1 | 1 | 0 | 0/0 | 1 | 0 | 0 |
|  | 2 | ♀ | 4 | 0 | 0 | 0 | 0 | 0 | 0/0 | 1 | 0 | 0 |
|  | 3 | ♀ | 2 | 0 | 0 | 1 | 0 | 0 | 0/1 | 0 | 0 | 0 |
| **Control3** | 1 | ♂ | 2 | 0 | 0 | 0 | 1 | 0 | 1/1 | 1 | 0 | 0 |
|  | 2 | ♀ | 3 | 0 | 0 | 0 | 0 | 0 | 0/0 | 0 | 0 | 0 |
|  | 3 | ♂ | 2 | 0 | 0 | 0 | 0 | 0 | 0/0 | 1 | 0 | 0 |
| **Exposure1** | 1 | ♂ | 3 | 0 | 0 | 1 | 0 | 0 | 1/1 | 1 | 1 (mantle/foot) | 0 |
|  | 2 | ♀ | 2 | 1 | 0 | 0 | 0 | 0 | 1/1 | 1 | 1 (mantle/foot) | 0 |
|  | 3 | ♂ | 2 | 0 | 0 | 0 | 0 | 0 | 0/1 | 1 | 1 (mantle/foot) | 0 |
| **Exposure2** | 1 | ♀ | 3 | 1 | 1 | 1 | 0 | 0 | 0/1 | 1 | 0 | Granulocytoma |
|  | 2 | ♀ | 2 | 1 | 0 | 0 | 0 | 1 | 1/1 | 1 | 1 (mantle/foot) | 0 |
|  | 3 | ♂ | 3 | 0 | 0 | 0 | 0 | 1 | 0/0 | 0 | 0 | 0 |
| **Exposure3** | 1 | ♂ | 3 | 0 | 0 | 1 | 0 | 0 | 0/1 | 1 | 1 (hepatopan.) | Gonadal regression |
|  | 2 | ♀ | 3 | 0 | 0 | 1 | 0 | 0 | 0/0 | 0 | 1 (hepatopan.) | 0 |
|  | 3 | ♀ | 3 | 1 | 0 | 1 | 0 | 0 | 0/1 | 1 | 1 (hepatopan.) | 0 |
