# Supplementary material for: Oxytetracycline-induced inflammatory process without oxidative stress in blue mussels Mytilus trossulus
Source: Environ Sci Pollut Res Int. 2023 Jun 10;30(33):80462–77. doi: 10.1007/s11356-023-28057-z (PMC10345040; doi:10.1007/s11356-023-28057-z)
Supplement: Supplementary file 3 — Supplementary file3 (DOCX 68 KB) [file 11356_2023_28057_MOESM3_ESM.docx]

**Supplementary 1a - the procedure for measuring enzyme activities:**

Alkaline phosphatase (ALP, EC 3.1.3.1) activity was determined using a commercially available test kit (R-R-A-pS-V-A 17-128, Sigma-Aldrich). ALP was detected using Malachite Green Detection System with phosphopeptide as a substrate at λ=650 (one unit of activity is equivalent to 1 nmol pNPP hydrolyzed per minute).

Asparaginase (ASP, EC 3.5.1.1) activity was determined using a commercially available test kit (MAK007, Sigma-Aldrich). ASP activity is detected by an enzyme assay, which results in a colorimetric (λ=570 nm) product, proportional to the aspartate generated. One unit of asparaginase is defined as the amount of enzyme that catalyzes the formation of 1.0 µmol of aspartate per minute at 25 ºC.

Phenoloxidase (PO, EC 1.14.18.1) activity was measured according to Munoz et al. (2006); 100 μl of haemolymph was transferred to a 96-well plate and then 50 μl of 10 mM L-DOPA solution was added. Activation of PO converts L-DOPA to dopachinone and then to dopachrome, which can be determined spectrophotometrically in Δt=5min at λ=480. One unit of activity (U) is defined as the amount of enzyme that causes an increase in absorbance of 0.001 at λ=480 nm within 5 min (Asokan et al. 1997).

Glutathione S-transferases (GSTs, EC 2.5.1.18) activity were determined spectrophotometrically by the method of Habig et al. (Habig 1974). The reaction mixture contained 100 mM phosphate-buffered saline buffer (pH 6.5), 100 mM 1-chloro-2, 4-dinitrobenzene (CDNB), and the reaction started by adding supernatant and 100 mM glutathione as substrate. The absorbance was measured for 5 min at 340 nm. GSTs activity were expressed in nmol/min/mg of total protein concentration.

The catalase (CAT, 1.11.1.6) activity was determined spectrophotometrically as a change in absorbance at 480 nm following reaction of H_2_O_2_ with a standard excess of 2 mM KMnO_4_ and spectrophotometrical detection of the residual KMnO_4_ (Kankofer, 2001). The enzyme activity was expressed in U/mg of the total protein concentration.

**Supplementary 1b - measurement of glutathione and antioxidant concentrations:**

The concentration of total glutathione (tGSH = glutathione disulphide GSSG + reduced glutathione GSH) in the cytosolic fraction was measured using a commercially available assay kit provided by Sigma-Aldrich (CS0260). Formation of 2-nitro-5-thiobenzoic acid and GSSG was assessed spectrophotometrically as a change in 412 nm absorbance at 1 min intervals for 5 min at 25 °C. The concentration of tGSH was converted to nmol/mg of the total protein concentration.

The TAC (Total Antioxidant Capacity) assay was measured using the Total Antioxidant Capacity Assay Kit (MAK187, Sigma-Aldrich). Concentrations of small molecule and protein antioxidants (e.g. tocopherols, carotenes, vitamin A, ubiquinols) were determined using Trolox, a water-soluble vitamin E analog serving as an antioxidant standard. The prepared samples were measured at 570 nm and the amount of TAC was normalized to the protein content of the samples and expressed in nmol/mg of total protein concentration.

Malondialdehyde (MDA) level was detected using the Lipid Peroxidation (MDA) Assay Kit (MAK085, Sigma-Aldrich) by the reaction of MDA with thiobarbituric acid (TBA) forming the amount of colorimetric product proportional to MDA content. Samples were pipetted on a 96-well microplate and absorbance was measured at 532 nm. The amount of MDA was normalized to the sample’s protein content and expressed in nmol/mg of total protein concentration.

Carbonyls (CBO) level was detected using the Protein Carbonyl Content Assay Kit (MAK094, Sigma-Aldrich). Carbonyl content was determined by the derivatization of protein carbonyl groups with 2,4-dinitrophenylhydrazine (DNPH) leading to the formation of stable dinitrophenyl (DNP) hydrazone adducts that are proportional to the present carbonyls. Absorbance was measured at 375 nm, and the amount of CBO was normalized to the sample protein content and expressed in nmol/mg of total protein concentration.

**Supplementary 1c - method of mitochondria isolation and measurement of aromatisation efficiency (AE)**

Mitochondria were isolated according to Schnaitman and Greenawalt (1968) with some modifications. Single bivalve soft tissue was homogenized in 1 ml of MSHE buffer (210 mM mannitol, 70 mM sucrose, 5 mM HEPES, 1 mM EGTA, and 0.5% BSA), pH 7.2 at 4 °C using a glass homogenizer. Homogenate was centrifuged at 800 RCF for 10 minutes at 4°C. The obtained supernatant was centrifuged again at 10000 RCF for 15 minutes at 4 °C and the pellet containing mitochondrial fraction was resuspended in MSHE buffer. Total mitochondrial protein content (mg/mL) was determined using the Lowry method. Aromatisation efficiency was determined in the mitochondrial fractions by the tritiated water release method as described in Shimizu et al. (1995) with some modifications (Hallmann et al., 2019).

Muñoz P, Meseguer J, Esteban MÁ (2006) Phenoloxidase activity in three commercial bivalve species. Changes due to natural infestation with Perkinsus atlanticus. Fish Shellfish Immunol. 20:12–19. <https://doi.org/10.1016/J.FSI.2005.02.002>

Asokan R, Arumugam M, Mullainadhan P (1997) Activation of prophenoloxidase in the plasma and haemocytes of the marine mussel Perna viridis Linnaeus. Dev Comp Immunol 21:1–12. <https://doi.org/10.1016/S0145-305X(97)00004-9>

Habig WH, Pabst MJ, Jakoby WB (1974) Glutathione S-transferases. The first enzymatic step in mercapturic acid formation. J Biol Chem 249:7130-9 PMID: 4436300.

Kankofer M (2001) Antioxidative defence mechanisms against reactive oxygen species in bovine retained and not-retained placenta: Activity of glutathione peroxidase, glutathione transferase, catalase and superoxide dismutase. Placenta 22:466–472. <https://doi.org/10.1053/plac.2001.0650>

Schnaitman C, Greenwalt JW. (1968) Enzymatic properties of the inner and outer membranes of rat liver mitochondria. J. Cell Biol. 1968;38: 158-175. doi: 10.1083/jcb.38.1.

Shimizu Y, Yarborough CP, Elger W, et al (1995) Inhibition of aromatase activity in human placental microsomes by 13-retro-antiprogestins. Steroids 60:234–8. https://doi.org/10.1016/0039-28X(94)00043-850 C

Hallmann A, Konieczna L, Swiezak J, et al (2019) Aromatisation of steroids in the bivalve Mytilus trossulus. PeerJ 7: e6953. <https://doi.org/10.7717/peerj.6953>
